# Risk factors for the development of hypermagnesemia in patients prescribed magnesium oxide: a retrospective cohort study

**DOI:** 10.1186/s40780-019-0133-7

**Published:** 2019-02-13

**Authors:** Eri Wakai, Kenji Ikemura, Hiroko Sugimoto, Takuya Iwamoto, Masahiro Okuda

**Affiliations:** 1 Department of Pharmacy, Mie University Hospital, 2-174, Edobashi, Tsu, Mie 514-8507 Japan; 2 Department of Clinical Pharmacy and Biopharmaceutics, Mie University Graduate School of Medicine, 2-174, Edobashi, Tsu, Mie 514-8507 Japan

**Keywords:** Magnesium oxide, Hypermagnesemia, Renal failure

## Abstract

**Background:**

Magnesium oxide (MgO), an antacid and laxative, is widely used in Japan to treat constipation and peptic ulcers. Because serum Magnesium (Mg) levels are elevated in elderly and/or patients with renal failure, its periodic monitoring is recommended for patients prescribed MgO, in order to prevent MgO-induced hypermagnesemia. However, there is little information regarding the factors contributing to the development of MgO-induced hypermagnesemia. In the present study, we retrospectively investigated the risk factors of hypermagnesemia in patients prescribed MgO.

**Methods:**

Data of 3258 patients hospitalized in Mie University Hospital between October 2015 and September 2017, who were prescribed MgO tablets, were extracted from the electronic medical records. According to the inclusion and exclusion criteria, 320 of the 3258 patients were enrolled in this study. Hypermagnesemia was defined as serum Mg levels ≥2.5 mg/dL (by the Common Terminology Criteria for Adverse Events version 4.0). Uni- and multivariate analyses were performed to identify risk factors for the development of hypermagnesemia in patients prescribed MgO using the following variables: age, estimated glomerular filtration rate, blood urea nitrogen levels, MgO dose, duration of MgO administration, and co-administrated proton pump inhibitors, H_2_ blocker (famotidine), vitamin D_3_ drugs, and diuretics.

**Results:**

Seventy-five patients out of 320 (23%) developed grade 1 and grade 3 hypermagnesemia, with the occurrence of grade 1 and grade 3 in 62 (19%) and 13 (4%) patients, respectively. Multivariate logistic regression analyses indicated 4 independent risk factors for hypermagnesemia comprising estimated glomerular filtration rate ≤ 55.4 mL/min (odds ratio (OR): 3.105, *P* = 0.001), blood urea nitrogen ≥22.4 mg/dL (OR: 3.490, *P* < 0.001), MgO dose ≥1650 mg/day (OR: 1.914, *P* = 0.039), and duration of MgO administration ≥36 days (OR: 2.198, *P* = 0.012). The occurrence rate of hypermagnesemia was elevated in accordance with these risk factors.

**Conclusions:**

These results suggest that a periodic monitoring of serum Mg levels is strongly recommended in MgO prescribed patients, especially in those with multiple risk factors for hypermagnesemia. The present findings provide useful information for the safe management of MgO therapy.

**Electronic supplementary material:**

The online version of this article (10.1186/s40780-019-0133-7) contains supplementary material, which is available to authorized users.

## Background

Magnesium oxide (MgO), a relatively cheap and safe antacid and laxative, is widely used in Japan for the treatment of constipation and peptic ulcers. In 2008, the cumulative number of patients treated with MgO was reported to be approximately 45 million in Japan, where includes approximately 120 million people [1]. On the other hand, severe hypermagnesemia cases including death and fatal symptoms such as cardiac conduction defect has been reported [2–6]. Therefore, the Ministry of Health, Labor and Welfare of Japan (MHLW) issued a letter to healthcare professionals for the periodic monitoring of serum Mg levels in patients with long-term use of MgO and/or elderly patients [1].

In the “PreAVOID Report” published by the Japanese Society of Hospital Pharmacists in 2015, amongst all drugs, discontinuation of MgO administration was most common [7]. Furthermore, most of the suggestions by pharmacists to physicians has been the discontinuation of MgO or switching to other laxatives, in patients with renal failure.

Nakamura et al. [8] reported that serum Mg levels were elevated in accordance with increased MgO dose in patients with renal failure. In elderly patients with MgO prescription, serum Mg levels were increased due to reduced renal function [9]. In addition, a recent retrospective study demonstrated that blood urea nitrogen (BUN) ≥ 22.5 mg/dL was a risk factor for developing hypermagnesemia in cancer patients prescribed MgO at palliative care hospital [10]. However, these reports comprise small number of cases with limited number of elderly and cancer patients. Although MHLW recommends the monitoring of serum Mg levels in patients with long-term use of MgO, the impact of prolonged duration of MgO administration on the development of hypermagnesemia remained to be clarified. Furthermore, there is limited information regarding the criteria for predicting the development of hypermagnesemia in patients prescribed MgO, including patients with normal renal function and younger patients.

In the present study, we retrospectively investigated risk factors for developing hypermagnesemia in patients prescribed MgO.

## Methods

### Patients and data collection

The data of patients (*n* = 3258) hospitalized in Mie University Hospital between October 2015 and September 2017, prescribed MgO tablet (Magmitt® Tab. 330 mg, Nichi-Iko Pharmaceutical Co., Ltd., Toyama, Japan), were extracted from the electronic medical records. Patients were excluded if they had missing data (*n* = 12), < 20 years of age (*n* = 35), and prescribed MgSO_4_ (*n* = 134). Moreover, because most patients who received powdered MgO (*n* = 215) were not able to intake dietary Mg, these patients were excluded to reduce potential bias related to confounding by reason for prescription on the analysis.

**Fig. 1:**
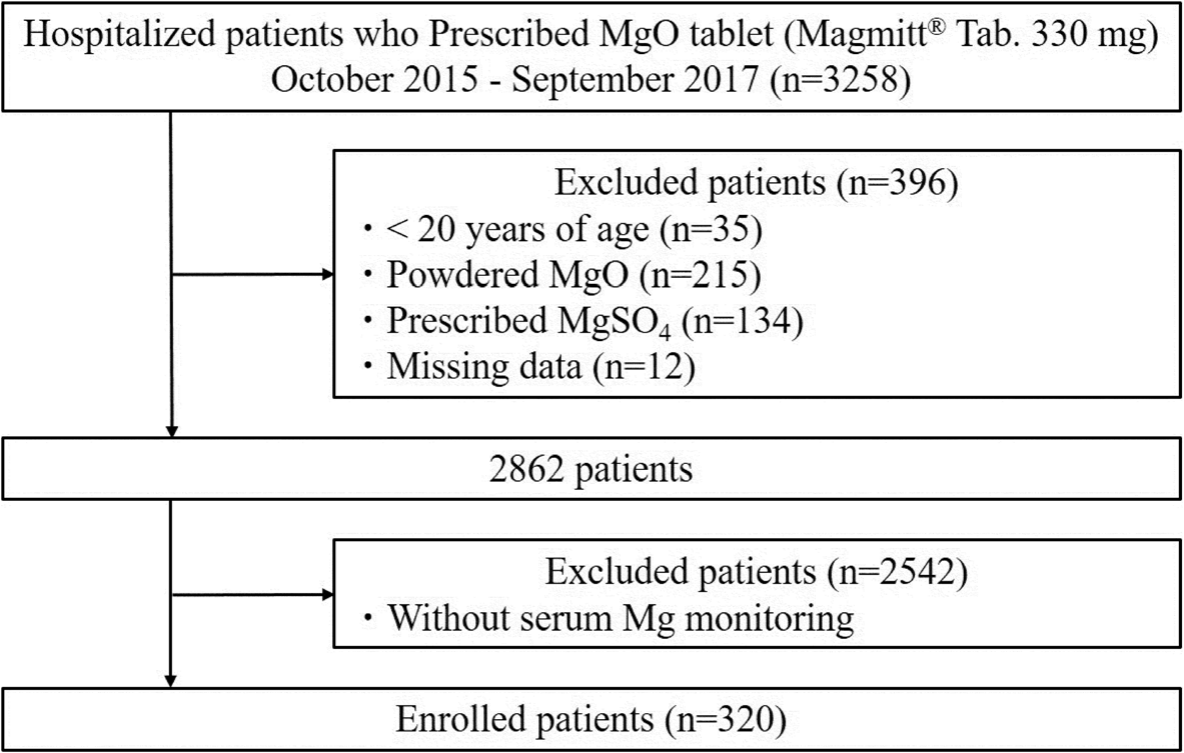
Flow chart of the patient selection

As shown in Fig. 1, a retrospective study was conducted in 320 patients with testing of serum Mg levels within 30 days of MgO administration. To avoid overestimation of serum creatinine (Scr) levels due to the influence of patients’ muscle mass, value of Scr < 0.6 mg/dL was substituted for Scr = 0.6 mg/dL [11], and estimated glomerular filtration rate (eGFR) was calculated using eGFR (mL/min/1.73 m^2^) = 194 × age^− 0.287^ × Scr^− 1.094^ × 0.739 (if female) [12]. eGFR (mL/min) = eGFR (mL/min/1.73 m^2^) × body surface area/1.73 (m^2^). Hypermagnesemia is defined as grade 1 if the serum Mg levels are ≥2.5 mg/dL (by the Common Terminology Criteria for Adverse Events version 4.0 (CTCAE)) [13]. In addition, co-administered drugs which may affect the serum Mg levels (VD_3_ and diuretics [14]) or cause potential interaction with MgO (PPIs or H_2_ blocker [15]) were investigated.

### Statistical analyses

Cut-off values of continuous variables for the development of hypermagnesemia (Grade ≥ 1) were determined by receiver operating characteristics (ROC) curve method with JMP® version 12.0.1 (SAS Institute Inc., Cary, NC, USA). Continuous variables were converted to dummy variables. Univariate analyses were performed to identify risk factors for development of hypermagnesemia (Grade ≥ 1) with following variables; age, eGFR, BUN, MgO dose, duration of MgO administration, co-administration of proton pump inhibitors (PPIs), H_2_ blocker (famotidine), vitamin D_3_ (VD_3_) drugs, and diuretics. In addition, multivariate analysis was conducted with variables, which was detected as *P* value < 0.20 in the univariate analyses, using the simultaneous force entry method. Statistical analyses were performed with IBM SPSS statistics for Windows version 23.0 (Armonk, NY, USA). Significance was established at a *P* value < 0.05.

## Results

### Patients’ characteristics

According to the inclusion and exclusion criteria, 320 of 3258 patients were enrolled. Patients’ characteristics are summarized in Table 1. 176 patients (55%) were female. The median age of patients was 42 (range, 20–95 years). The median eGFR and BUN were 75.7 mL/min (range, 3.4–158.4) and 23.4 mg/dL (range, 6.2–189.8), respectively. The median dose of MgO was 990 mg/day (range, 330–2970). The median duration of MgO administration was 52 days (range, 1–348). Moreover, the number of patients co-administered with PPI, famotidine, VD_3_ drugs, and diuretics were 133 (46%), 19 (6%), 23 (7%), and 16 (5%) respectively.

**Table 1:** Characteristics of the patients enrolled in the study

Characteristics	
Number of patients	320
Female	176 (55)
Age (years)	42 [20–95]
Body weight (kg)	54.3 [26.0–101.2]
eGFR (mL/min)	75.7 [3.4–158.4]
BUN (mg/dL)	23.4 [6.2–189.8]
MgO dose (mg/day)	990 [330–2970]
Duration of MgO administration (days)	52 [1–348]
Co-administrated drugs	
PPIs	133 (46)
H_2_ blocker (famotidine)	19 (6)
VD_3_	23 (7)
Diuretics	16 (5)

Values are presented as median [range] or number (%)

*BUN* blood urea nitrogen, *eGFR* estimated glomerular filtration rate, *MgO* magnesium oxide, *PPIs* proton pump inhibitors, *VD*_*3*_ vitamin D_3_

### Occurrence rate and severity of hypermagnesemia in patients with MgO

The number of patients with hypermagnesemia are summarized in Table 2. 75 of 320 patients (23%) developed hypermagnesemia. Grade 1 and grade 3 of hypermagnesemia were observed in 62 patients (19%) and 13 patients (4%), respectively. More severe hypermagnesemia (≥ Grade 4) was not observed in any patient.

**Table 2:** Occurrence rate and severity of hypermagnesemia in patients prescribed MgO

	Grade				
Hypermagnesemia	0	1	3	4	5
	245 (77)	62 (19)	13 (4)	0 (0)	0 (0)

Values are presented as number (%)

Hypermagnesemia was evaluated according to CTCAE ver. 4.0

### Risk factors for the development of hypermagnesemia

Although the multi-collinearity among variables was evaluated, strong correlations (|correlation coefficient: r | > 0.7) were not observed. Logistic regression analysis was conducted to investigate the risk factors for the development of hypermagnesemia in patients prescribed MgO (Table 3). The cut-off values (area under the ROC curve: AUC) of age, eGFR, BUN, MgO dose, and duration of MgO administration were 68 years (0.64), 55.4 mL/min (0.70), 22.4 mg/dL (0.58), 1650 mg/day (0.72), and 36 days (0.58), respectively. Univariate analysis indicated that risk factors significantly contributing to the development of hypermagnesemia were age ≥ 68 years (odds ratio (OR): 2.548, *P* < 0.001), eGFR ≤55.4 mL/min (OR: 4.564, *P* < 0.001), BUN ≥22.4 mg/dL (OR: 4.793, *P* < 0.001), MgO dose ≥1650 mg/day (OR: 2.004, *P* = 0.011), and duration of MgO administration ≥36 days (OR: 2.074, *P* = 0.009). Multivariate analysis revealed that independent risk factors for the development of hypermagnesemia were eGFR ≤55.4 mL/min (OR: 3.105, *P* = 0.001), BUN ≥22.4 mg/dL (OR: 3.490, *P* < 0.001), MgO dose ≥1650 mg/day (OR: 1.914, *P* = 0.039), and duration of MgO administration ≥36 days (OR: 2.198, *P* = 0.012). Moreover, these significant variables were also detected as independent risk factors in multivariate analyses using stepwise forward and backward selection methods.

**Table 3:** Logistic regression analyses for risk factors of hypermagnesemia in patients prescribed MgO

Variables	Univariate analysis			Multivariate analysis		
	Odds ratio	95% CI	*P* value	Odds ratio	95% CI	*P* value
Age ≥ 68 years	2.548	1.498–4.330	< 0.001	1.710	0.937–3.123	0.081
eGFR ≤55.4 mL/min	4.564	2.624–7.938	< 0.001	3.105	1.642–5.872	0.001
BUN ≥22.4 mg/dL	4.793	2.580–8.940	< 0.001	3.490	1.762–6.911	< 0.001
MgO dose ≥1650 mg/day	2.004	1.175–3.418	0.011	1.914	1.034–3.542	0.039
Duration of MgO administration ≥36 days	2.074	1.201–3.580	0.009	2.198	1.190–4.060	0.012
Co-administrated drugs						
PPIs	1.061	0.629–1.791	0.825			
H_2_ blocker (famotidine)	1.999	0.757–5.275	0.162	0.538	0.175–1.657	0.280
VD_3_ drugs	1.417	0.357–5.620	0.620			
Diuretics	1.831	0.744–4.504	0.188	1.348	0.471–3.863	0.578

*BUN* blood urea nitrogen, *CI* confidence interval, *eGFR* estimated glomerular filtration rate, *MgO* magnesium oxide, *PPIs* proton pump inhibitors, *VD*_*3*_ vitamin D_3_

### Relationship between the number of risk factors and occurrence ratio of hypermagnesemia

**Fig. 2:**
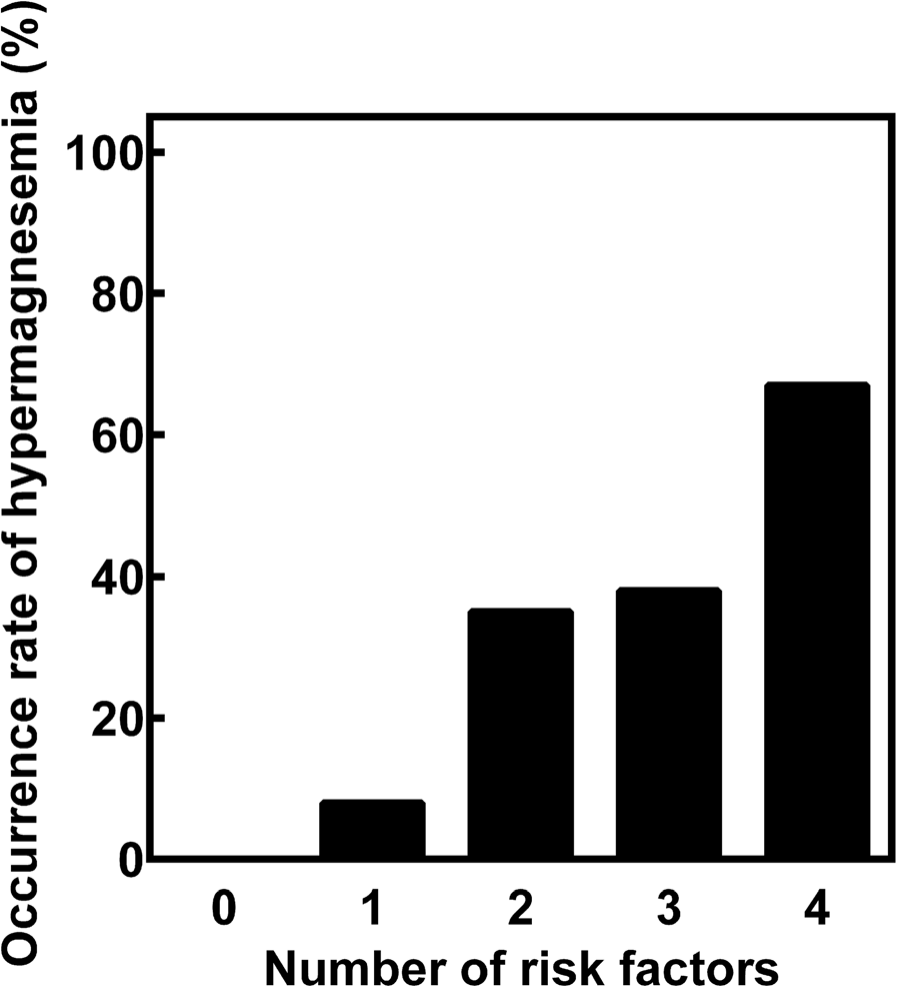
Relationship between the number of risk factors and occurrence rate of hypermagnesemia

Based on the results shown in Table 3, we analyzed the relationship between the different risk factors and the occurrence ratio of hypermagnesemia, following administration of MgO (Fig. 2). The ratio of hypermagnesemia in each group divided by the number of risk factors in ascending order was 0% (0/45), 10% (10/105), 33% (32/97), 38% (21/55), and 67% (12/18), respectively.

## Discussion

Little is known regarding factors distinguishing the development of hypermagnesemia in patients prescribed MgO. Although MHLW recommends the monitoring of serum Mg levels in patients with long-term use of MgO [1], the relationship between duration of MgO administration and hypermagnesemia is unclear. Our study is the first to demonstrate that decreased renal function as well as prolonged duration of MgO administration could increase the risk of hypermagnesemia in patients prescribed MgO.

Moreover, multivariate analysis indicated that MgO dose ≥1650 mg/day was a significant independent risk factor for hypermagnesemia in patients prescribed MgO (Table 3). Previous studies demonstrated that elevated serum Mg levels were observed in patients with severe renal failure (eGFR < 15 mL/min), upon administration of MgO dose ≥1000 mg/day [8, 16]. Since our study was conducted in patients with normal and decreased renal function (Table 1), the differences in the cut-off values for renal function could be attributed to the discrepancy of Mg dose between studies. Therefore, hypermagnesemia, following MgO treatment, should be developed not only in patients with decreased renal function but also in patients with normal renal function, in accordance with increased dose of MgO.

As shown in Table 3, BUN ≥22.4 mg/dL and eGFR ≤55.4 mL/min are risk factors for hypermagnesemia in patients prescribed MgO. Nakashima et al. [10] demonstrated that BUN (≥ 22.5 mg/dL) was a significant risk factor of hypermagnesemia in patients with MgO administration. The results described in the present study are comparable to those of Nakamura et al. [8], where serum Mg levels were elevated in patients prescribed MgO (especially in patients with renal failure). Therefore, monitoring of serum Mg levels should be necessary in the patients with decreased renal function.

Although serum Mg levels are known to be increased in elderly patients with MgO administration [17, 18], age was not a significant risk factor for hypermagnesemia in our present study (Table 3). In general, eGFR is negatively correlated with age, suggesting decreased renal function in the elderly [19]. In the present study, we also confirmed the negative correlation between eGFR and age (*r* = − 0.05, *P* < 0.001, Additional file 1: Figure S1). Although age was not a significant risk factor for hypermagnesemia in the present study, decreased renal function with age should be a criterion for the development of hypermagnesemia.

On the other hand, serum Mg level is known to be elevated in patients with the treatment of lithium therapy, and with hypothyroidism and Addison disease [20]. Since there were few patients with lithium therapy (*n* = 3), hypothyroidism (*n* = 11), and Addison disease (*n* = 0), we could not analyze the effect of these factors on the development of hypermagnesemia. However, we speculate that these factors could have little influence on the development of hypermagnesemia in patients with MgO because the development of hypermagnesemia in patients with these factors was not observed.

The MHLW recommended monitoring of serum Mg levels in patients treated with MgO [1]. However, there is limited information about monitoring serum Mg levels in patients with MgO administration, in clinical settings. In the present study, only 11% (320/2862) of the patients prescribed MgO were subjected to serum Mg measurement, indicating that the monitoring of serum Mg levels in patients with MgO therapy is exceptionally low (Fig. 1). To explore the factors influencing serum Mg monitoring, patients’ characteristics were compared with and without serum Mg measurement (Additional file 2: Table S1). Renal function, in the patients measured for Mg levels, was significantly lower than that in patients where Mg levels were unmeasured. In addition, duration of MgO administration in the patients measured for Mg levels was significantly longer than that in patients where Mg levels were not measured. Therefore, these finding suggested that physicians, at least, in part, should be mindful to serum Mg monitoring in patients with decreased renal function and/or with long-term administration of MgO, which is similar to the recommendations by MHLW.

However, among 2542 patients without serum Mg monitoring, 66% (1676/2542) of patients had at least one risk factor of hypermagnesemia associated with MgO administration (Additional file 3: Figure S2). Furthermore, 1% (16/2542) of the patients without serum Mg monitoring had four risk factors of hypermagnesemia. Surprisingly, among 320 patients with serum Mg monitoring, severe hypermagnesemia, accompanied with unconsciousness, was observed in patients with four risk factors. Because the occurrence rate of hypermagnesemia was elevated in accordance with increased number of risk factors (Fig. 2), development of hypermagnesemia would be overlooked in the patients without serum Mg monitoring.

Our present study has several limitations. First, it was difficult to exclude the potential effects of unknown confounders. Second, patients’ adherence to MgO therapy could not be monitored, although it is assumed that patients’ adherence to the medication would have been good, as it was checked by a nurse in the hospital. Therefore, a prospective study should be conducted to evaluate risk factors of hypermagnesemia in patients with MgO therapy.

## Conclusions

Our study was the first to demonstrate that decreased renal function (eGFR ≤55.4 mL/min and BUN ≥22.4 mg/dL) and/or prolonged duration of MgO administration (≥ 36 days), and increased MgO dose (≥ 1650 mg/day) could increase the risk of hypermagnesemia in patients prescribed MgO. These findings suggested that a periodical monitoring of serum Mg levels is recommended in patients prescribed MgO, especially in those with multiple risk factors for developing hypermagnesemia. The present findings provide useful information for the achievement of appropriate use of MgO.

## Additional files

Additional file 1: **Figure S1:** Correlation between eGFR and age in patients prescribed MgO and tested for serum Mg levels (*n* = 320). Statistical analysis was performed using Spearman correlation coefficient. Each point represents a patient. (PDF 65 kb)

Additional file 2: **Table S1:** Comparison of patients’ characteristics with and without serum Mg measurement. Values are presented as median [range] or number (%). BUN blood urea nitrogen, eGFR estimated glomerular filtration rate, MgO magnesium oxide, PPIs proton pump inhibitors, VD_3_ vitamin D_3_. Statistical analyses were performed using chi-square test or Mann-Whitney U-test. (PDF 199 kb)

Additional file 3: **Figure S2:** Distribution of patients classified by the number of suggested risk factors for hypermagnesemia in patients without serum Mg monitoring (*n* = 2542). Values are presented as number (%). (PDF 78 kb)

## Abbreviations

AUC: Area under the ROC curve
BUN: Blood urea nitrogen
CTCAE: Common Terminology Criteria for Adverse Events
eGFR: estimated glomerular filtration
Mg: Magnesium
MgO: Magnesium oxide
MHLW: Ministry of Health, Labor and Welfare of Japan
PPIs: Proton pomp inhibitors
ROC: Receiver operating characteristic
Scr: Serum creatinine
VD_3_: Vitamin D_3_
