# Supplementary material for: Risk factors for the development of hypermagnesemia in patients prescribed magnesium oxide: a retrospective cohort study
Source: J Pharm Health Care Sci. 2019 Feb 13;5:4. doi: 10.1186/s40780-019-0133-7 (PMC6373027; doi:10.1186/s40780-019-0133-7)
Supplement: Supplementary file 1 — Figure S1: Correlation between eGFR and age in patients prescribed MgO and tested for serum Mg levels (n = 320). Statistical analysis was performed using Spearman correlation coefficient. Each point represents a patient. (PDF 65 kb) [file 40780_2019_133_MOESM1_ESM.pdf]

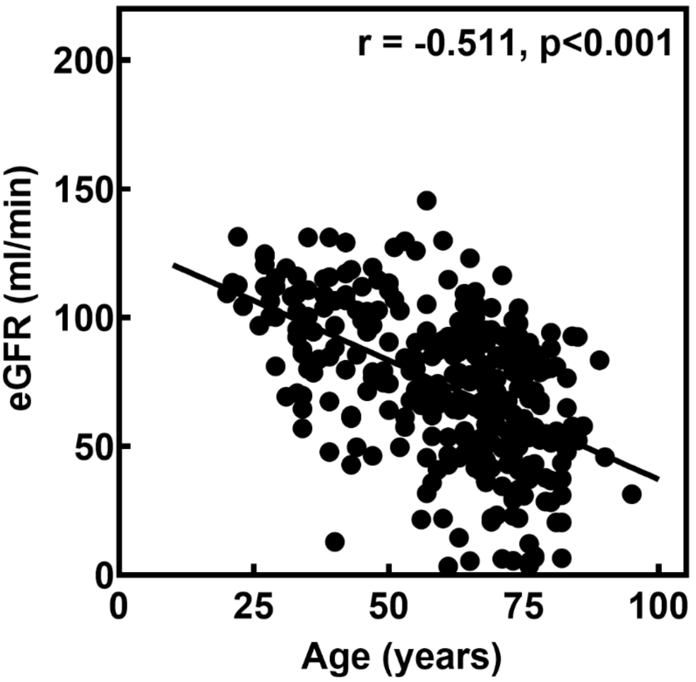

Additional figure 1. Correlation between eGFR and age in patients prescribed MgO and tested for serum Mg levels (n=320).

Statistical analysis was performed using Spearman correlation coefficient. Each point represents a patient.
