# Supplementary material for: Risk factors for the development of hypermagnesemia in patients prescribed magnesium oxide: a retrospective cohort study
Source: J Pharm Health Care Sci. 2019 Feb 13;5:4. doi: 10.1186/s40780-019-0133-7 (PMC6373027; doi:10.1186/s40780-019-0133-7)
Supplement: Supplementary file 2 — Table S1: Comparison of patients’ characteristics with and without serum Mg measurement. Values are presented as median [range] or number (%). BUN blood urea nitrogen, eGFR estimated glomerular filtration rate, MgO magnesium oxide, PPIs proton pump inhibitors, VD3 vitamin D3. Statistical analyses were performed using chi-square test or Mann-Whitney U-test. (PDF 199 kb) [file 40780_2019_133_MOESM2_ESM.pdf]

Additional table 1. Comparison of patients' characteristics with and without serum Mg measurement.

Characteristics	All patients (n=2862)	Unmeasured (n=2542)	Measured (n=320)	*P* value
Female	1600 (56)	1464 (58)	176 (55)	0.377
Age (years)	66 [20–104]	66 [20–104]	42 [20–95]	0.709
Body weight (kg)	55.6 [26.0–123.4]	55.7 [26.4–123.4]	54.3 [26.0–101.2]	0.201
eGFR (mL/min)	75.7 [3.4–158.4]	75.9 [3.5–158.4]	75.1 [3.4–145.4]	0.493
BUN (mg/dL)	14.8 [2.5–189.8]	14.3 [2.5–104.2]	23.4 [6.2–189.8]	<0.001
MgO dose (mg/day)	990 [330–2970]	990 [330–2970]	990 [330–2970]	0.042
Duration of MgO administration (days)	21 [1–348]	20 [1–364]	52 [1–348]	<0.001
Co-administrated drugs				
PPIs	1303 (45)	1170 (46)	133 (46)	0.131
Famotidine	102 (6)	143 (5)	19 (6)	0.820
VD_3 drugs	204 (7)	181 (7)	23 (7)	0.965

Values are presented as median [range] or number (%).

BUN blood urea nitrogen, *eGFR* estimated glomerular filtration rate, *MgO* magnesium oxide, *PPIs* proton pump inhibitors, VD_3 vitamin D_3. Statistical analyses were performed using chi-square test or Mann-Whitney U-test.
