# Supplementary material for: Risk factors for the development of hypermagnesemia in patients prescribed magnesium oxide: a retrospective cohort study
Source: J Pharm Health Care Sci. 2019 Feb 13;5:4. doi: 10.1186/s40780-019-0133-7 (PMC6373027; doi:10.1186/s40780-019-0133-7)
Supplement: Supplementary file 3 — Figure S2: Distribution of patients classified by the number of suggested risk factors for hypermagnesemia in patients without serum Mg monitoring (n = 2542). Values are presented as number (%). (PDF 78 kb) [file 40780_2019_133_MOESM3_ESM.pdf]

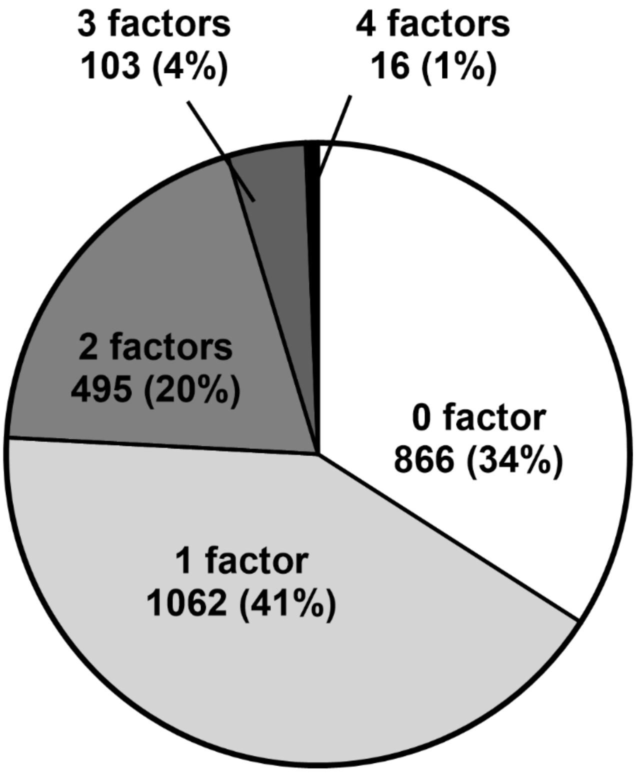

Additional figure 2. Distribution of patients classified by the number of suggested risk factors for hypermagnesemia in patients without serum Mg monitoring (n=2542).

Values are presented as number (%).
